# Supplementary material for: Optics miniaturization strategy for demanding Raman spectroscopy applications
Source: Nat Commun. 2024 Apr 8;15:3049. doi: 10.1038/s41467-024-47044-7 (PMC11001912; doi:10.1038/s41467-024-47044-7)
Supplement: Supplementary file 1 — Supplementary Information [file 41467_2024_47044_MOESM1_ESM.pdf]

Supplementary Materials for

Optics miniaturization strategy for demanding Raman spectroscopy applications

Oleksii Ilchenko[1,2], Yurii Pilhun[2,3], Andrii Kutsyk[2,3,4], Denys Slobodianiuk[3,5], Yaman Goksel[1], Elodie Dumont[1], Lukas Vaut[1], Chiara Mazzoni[1], Lidia Morelli[1], Sofus Boisen[2], Konstantinos Stergiou[2], Yaroslav Aulin[2], Tomas Rindzevicius[1], Thomas Emil Andersen[6], Mikael Lassen[7], Hemanshu Mundhada[8], Christian Bille Jendresen[8], Peter Alshede Philipsen[9], Merete Hædersdal[9], Anja Boisen[1]

Correspondence to: olil@lightnovo.com

This PDF file includes:

Supplementary Text
Figs. S1 to S33
Tables S1 to S6
Captions for Movie S1
Captions for Data S1 to S7
References

Supplementary Text

S1 “Mode hop” deconvolution.

Blurred signal can be written as convolution of PSF and real (ideal) signal:

$$\tilde{f}(x) = \int_{-\infty}^{\infty} psf(x-\chi)f(\chi)d\chi = psf * f \qquad (1)$$

In matrix form

$$\tilde{f} = \hat{A}_{psf} f = \hat{A}_f \cdot psf \qquad (2)$$

The above equation represents symmetry properties of convolution. It allows us to estimate PSF using ideal and blurred signals. The obtained PSF can be used for the deblurring of measured signals.

Since measured blurred signal contains noise, the solution should be found in sense of least squares. The PSF estimation problem can be rewritten as classical least squares problem:

$$\mathrm{psf} = \mathrm{argmin}_{\mathbf{x}} \|\mathbf{y}\text{-}\hat{\mathbf{A}}\mathbf{x}\|_2^2 \qquad (3)$$

To constrain result to be non-negative, NNLS should be used:

$$\mathrm{psf} = \mathrm{argmin}_{\mathbf{x}} \|\mathbf{y}\text{-}\hat{\mathbf{A}}\mathbf{x}\|_2^2 \mathit{subject\ to}\ \mathbf{x} \geq 0 \qquad (4)$$

These problems are ill-posed and regularization steps are required. We considered several types of regularization[1]:

1) L1 regularization

$$\mathbf{psf} = \mathrm{argmin}_{\mathbf{x}} \|\mathbf{y}\text{-}\hat{\mathbf{A}}\mathbf{x}\|_2^2 + \lambda^2\ \|\mathbf{x}\|_1^2 \qquad (5)$$

2) L2 regularization (robust regression)

$$\mathbf{psf} = \mathrm{argmin}_{\mathbf{x}} \|\mathbf{y}\text{-}\hat{\mathbf{A}}\mathbf{x}\|_2^2 + \lambda^2\ \|\mathbf{x}\|_2^2 \qquad (6)$$

3) L2 smooth regularization

$$\mathbf{psf} = \mathrm{argmin}_{\mathbf{x}} \|\mathbf{y}\text{-}\hat{\mathbf{A}}\mathbf{x}\|_2^2 + \lambda^2 \|\hat{\mathbf{D}}_2\mathbf{x}\|_2^2 \qquad (7)$$

Here $\hat{\mathbf{D}}_2$ is a second derivative matrix.

When the ‘ideal’ reference channel spectrum is available, the ‘mod hop’ deconvolution algorithm is the following:

1) Considering the reference channel spectrum at each step as blurred one, we can obtain PSF using the ‘ideal’ reference spectrum.
2) Data channel spectrum is deblurred using the obtained PSF.

We used the same value of the regularization parameter $\lambda = 0.1$. As the example, the ‘ideal’ reference channel spectrum (Fig. S2) and blurred reference channel spectrum (Fig. S3) were used to estimate the PSF (Fig. S4). The deblurred data channel spectrum is shown in Fig. S5. We used different techniques of regularization. The best result is obtained for L2 smooth regularization (Eq. 7)
The same procedure were applied to the time map (Fig. S6).

S2. Spectral deblurring

Diamond peak at 1332 cm^{-1} (Fig. S7) was used as for the estimation of PSF. Raman spectrum of diamond was baseline corrected using asymmetrical least squares[2] (Fig. S8). Parameters used for baseline estimation is the following: $\lambda = 10^4, p = 0.001$. The obtained PSF is shown in Fig. S9.

We used L2 nonnegative regularization (Eq. 6). FISTA algorithm implemented in IRTools[3] was used to solve this problem. Raman spectrum of toluene was used to test this deconvolution approach (Fig. S10). Background was estimated using asymmetric least squares with $\lambda = 10^5, p = 0.001$. The obtained results are shown in Fig. S11. It

worth to be noted that background correction is a crucial step. Otherwise, additional artefacts might occur. As it can be seen from the Fig. S12, such approach allows increasing spectral resolution in 1.5 times.

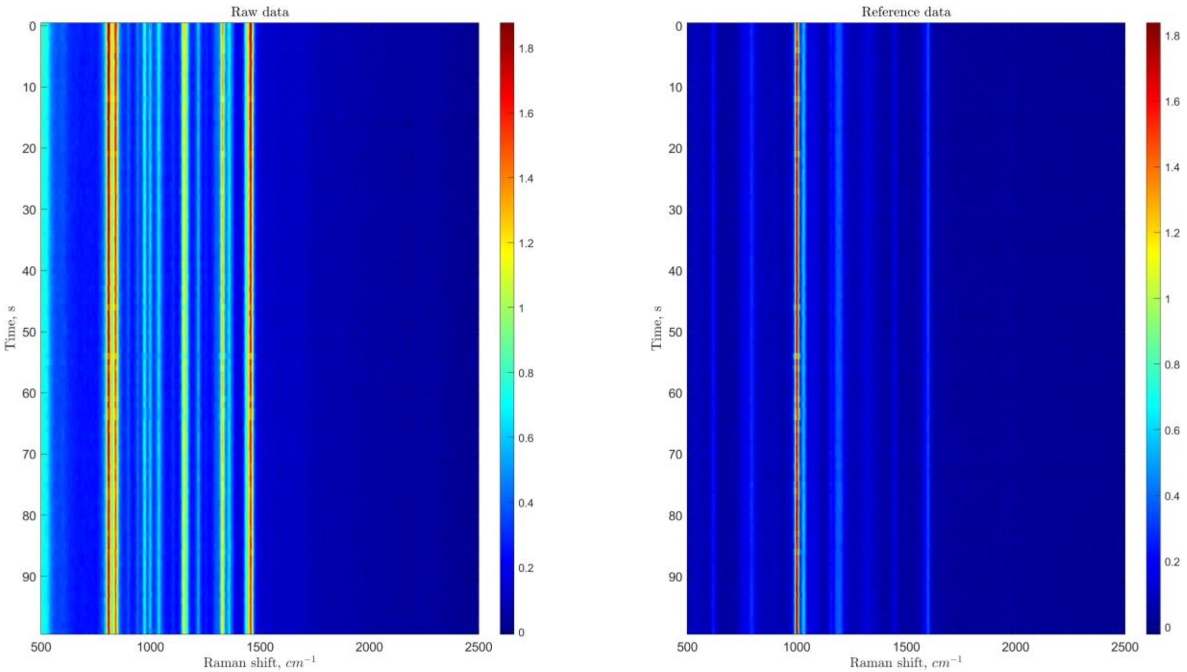

Fig. S1. Raw data and reference spectra.

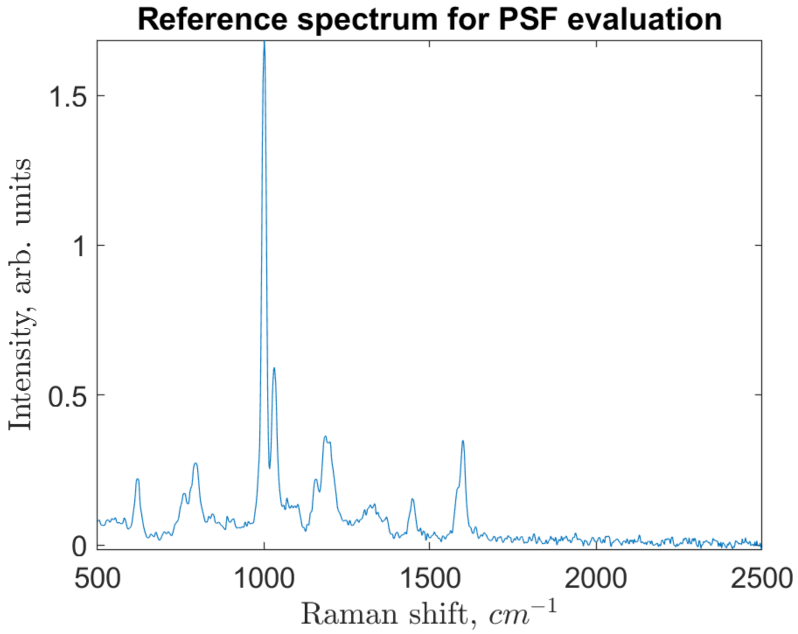

Fig. S2. Reference channel spectrum used as ideal.

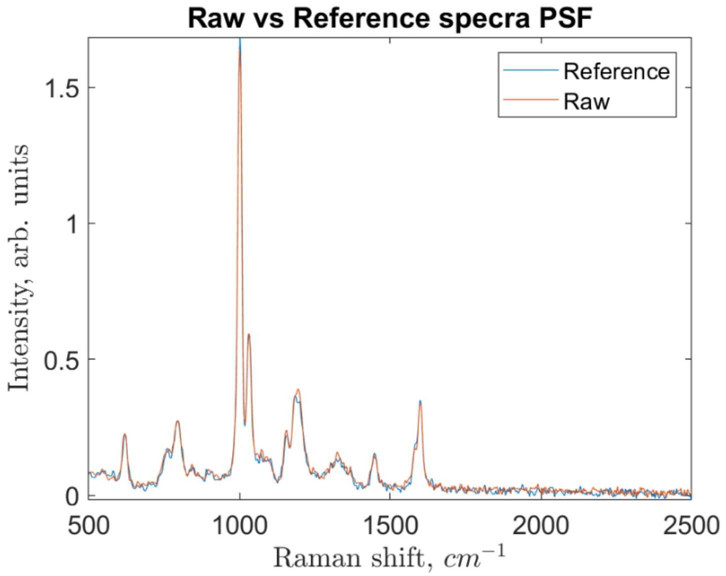

Fig. S3. ‘Raw’ and ‘ideal’ reference channel spectra

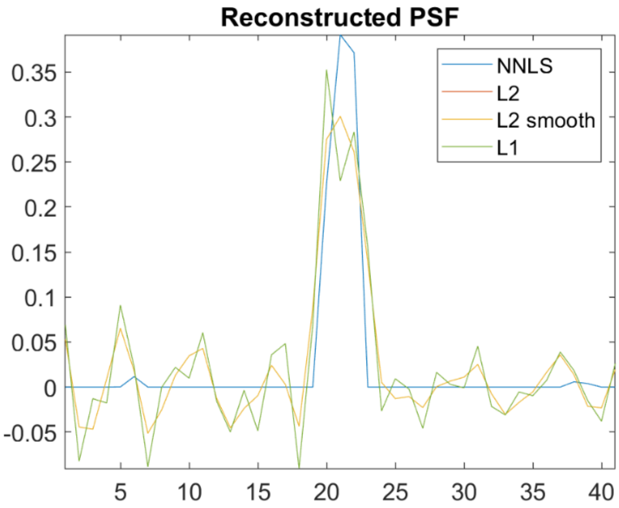

Fig.S4. The estimated PSF.

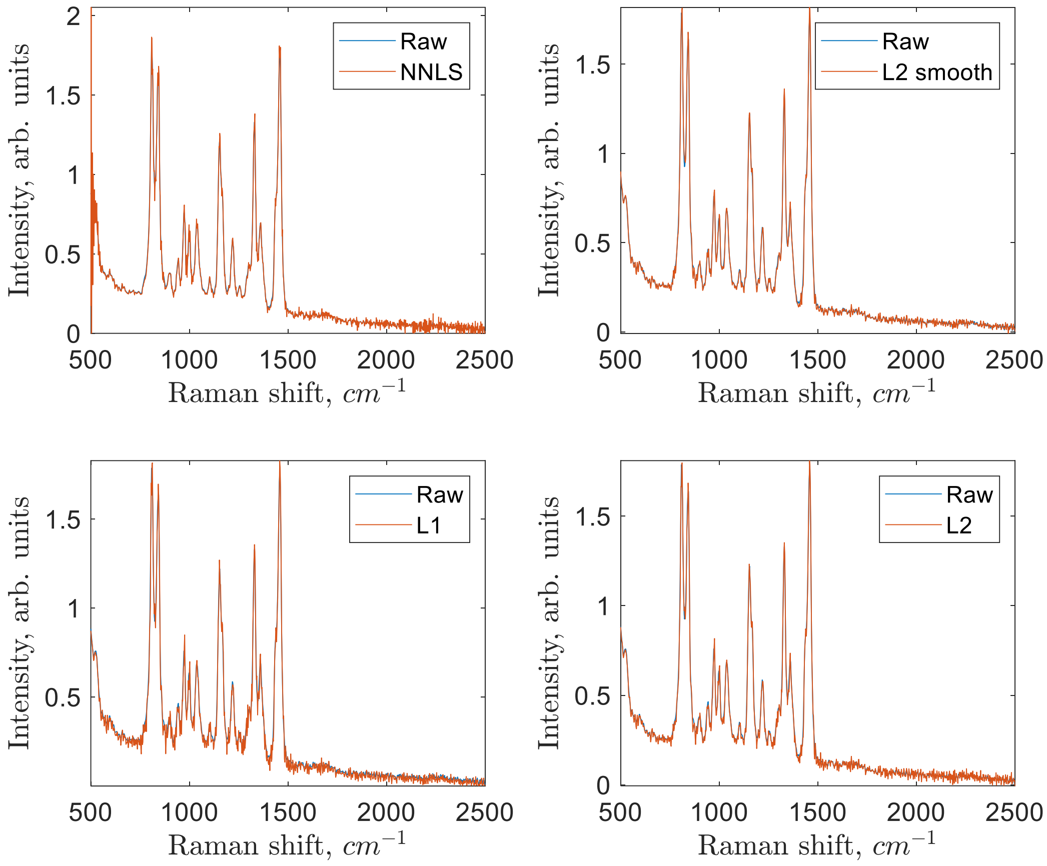

Fig. S5. Deblurred vs. Raw data channel spectra

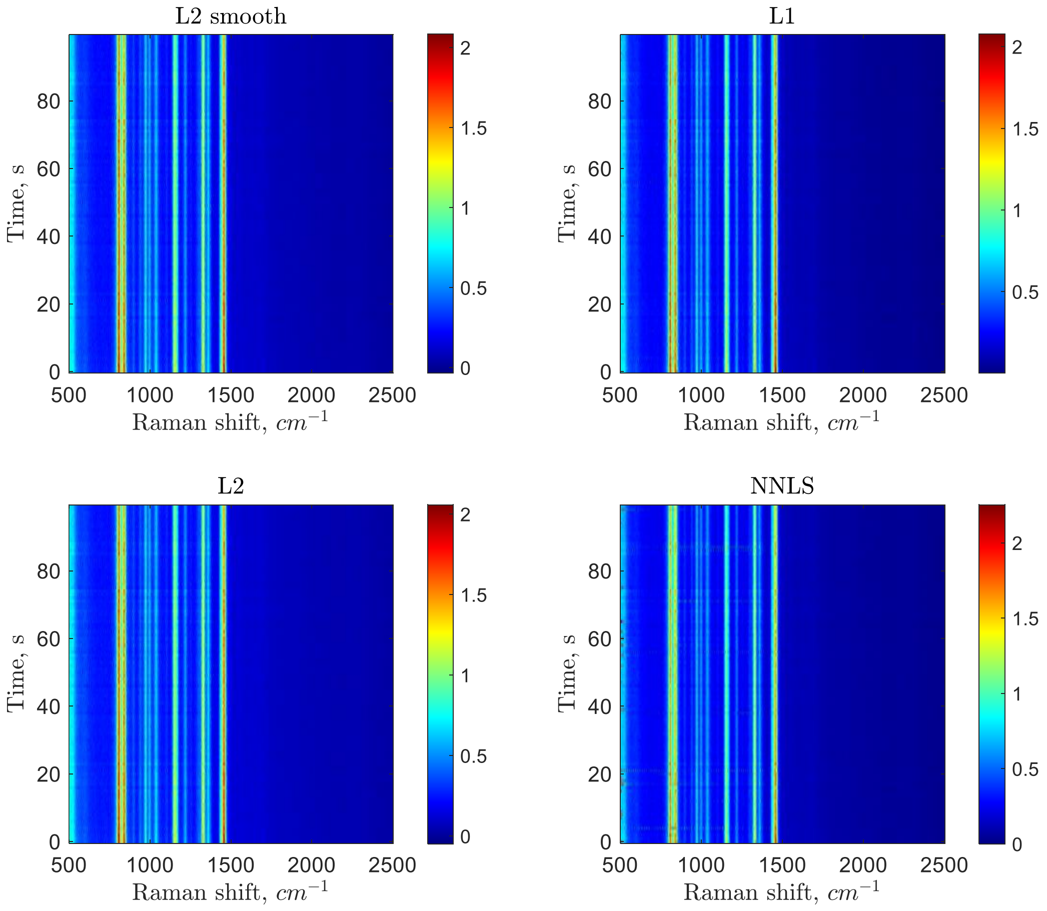

Fig. S6. Deblurred time maps

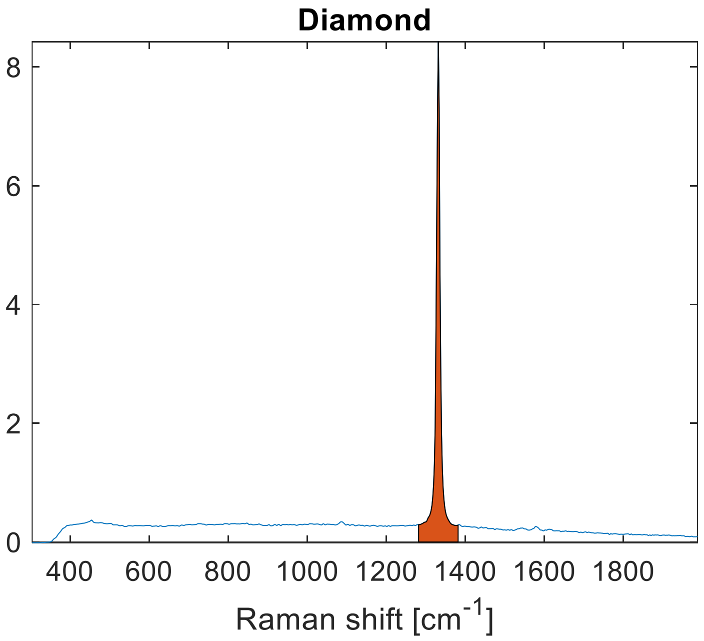

Fig. S7. Raman spectrum of diamond. The highlighted area is used for the PSF estimation.

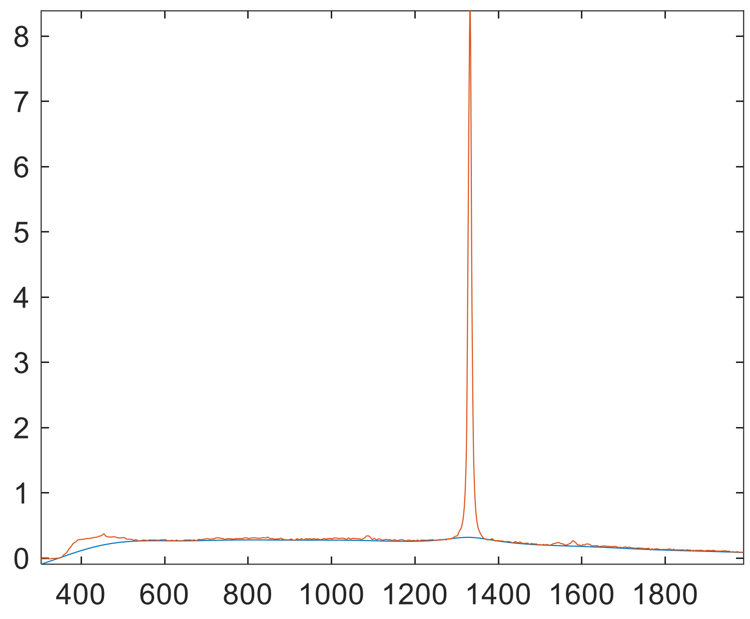

Fig. S8. The calculated baseline.

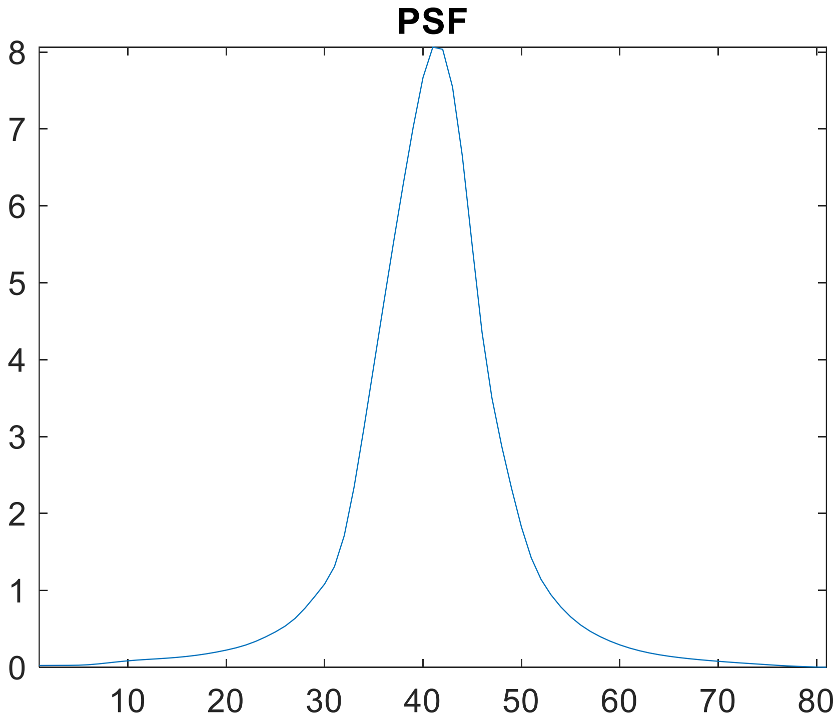

Fig. S9. The estimated PSF.

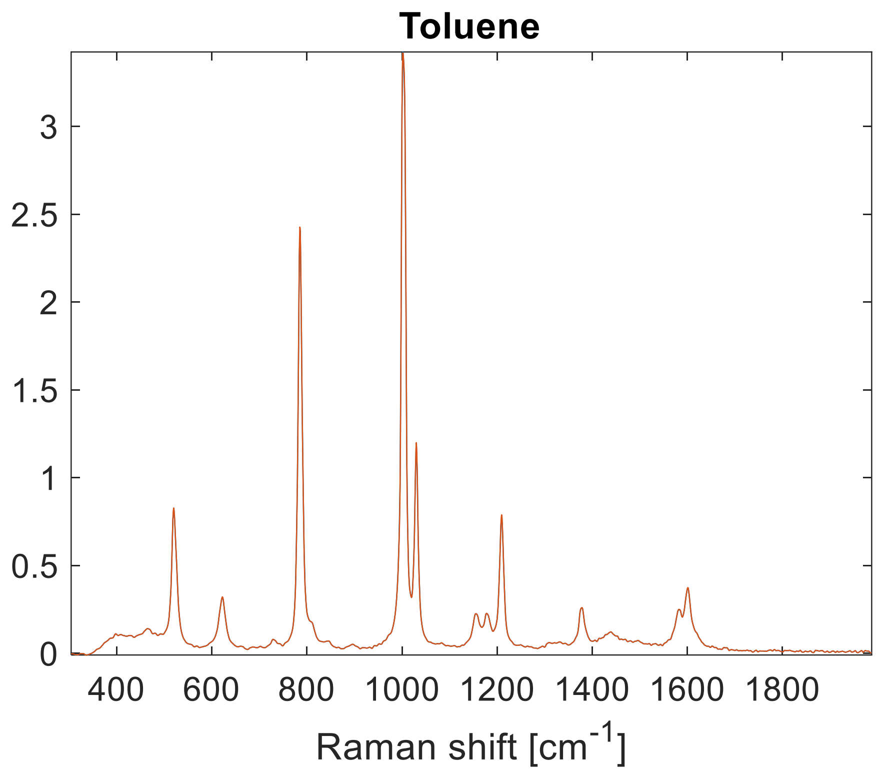

Fig. S10. Raman spectrum of toluene

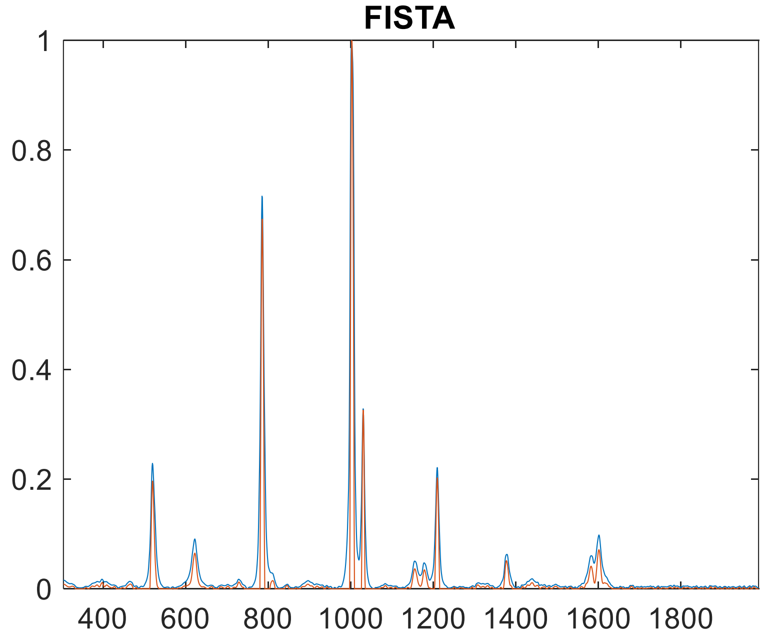

Fig.S11. Raw and deblurred spectra of toluene.

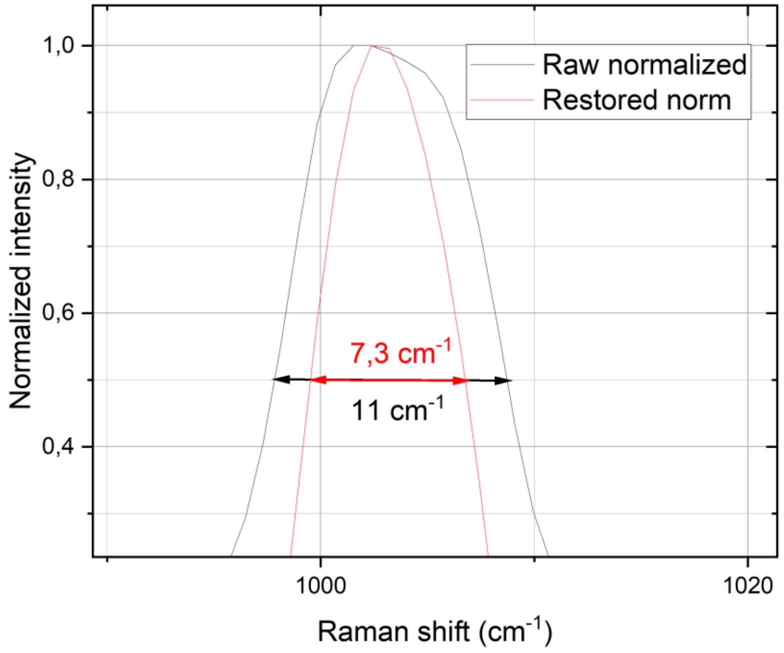

Fig. S12. Peak width at half height for the raw and deblurred spectra. They were peak height normalized.

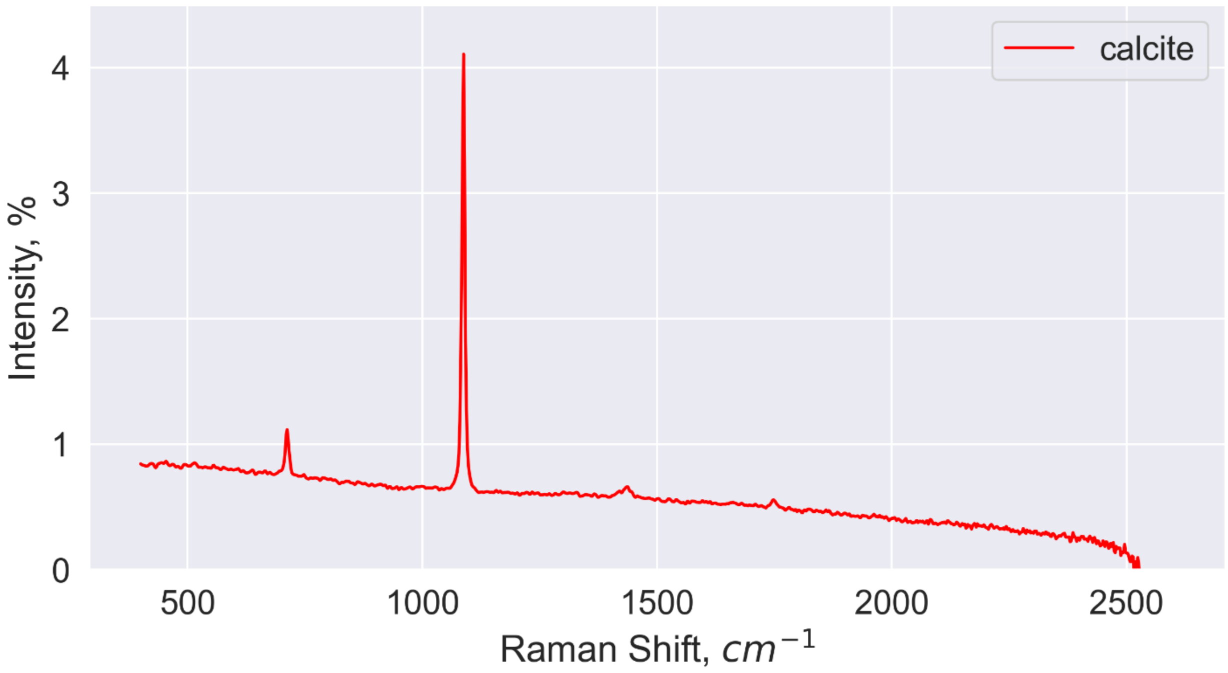

Fig. S13. Raman spectrum of calcite reference sample.

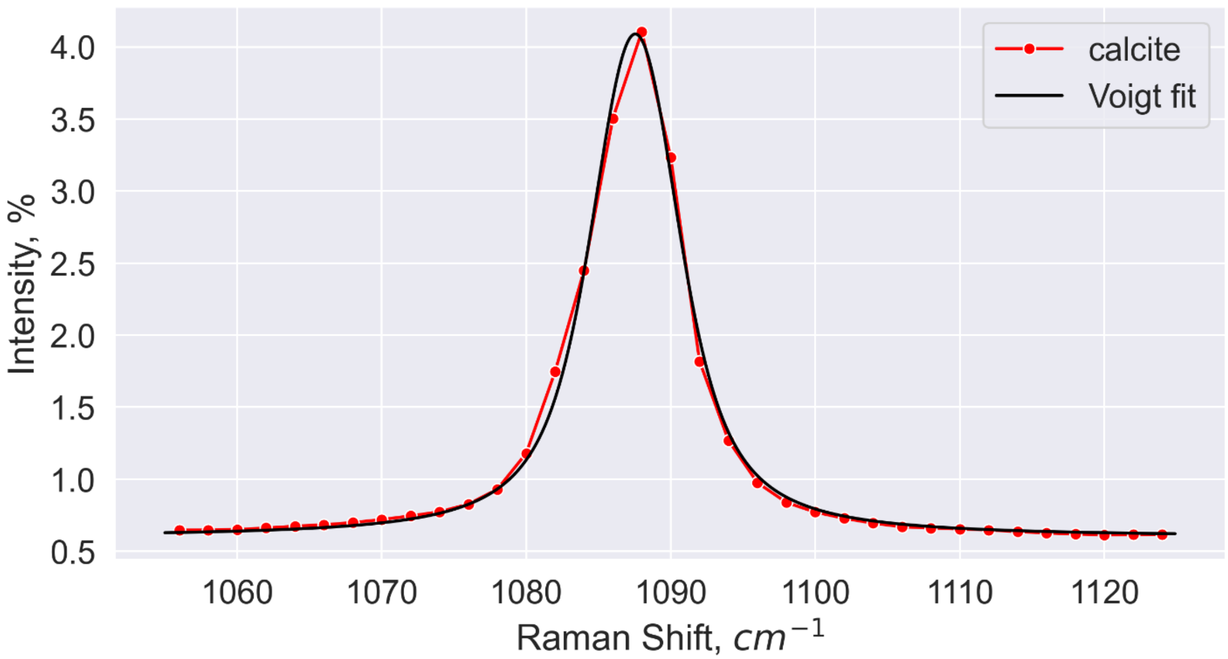

Fig. S14. Raman spectrum of calcite 1085cm^{-1} line and Voigt fit of the line.

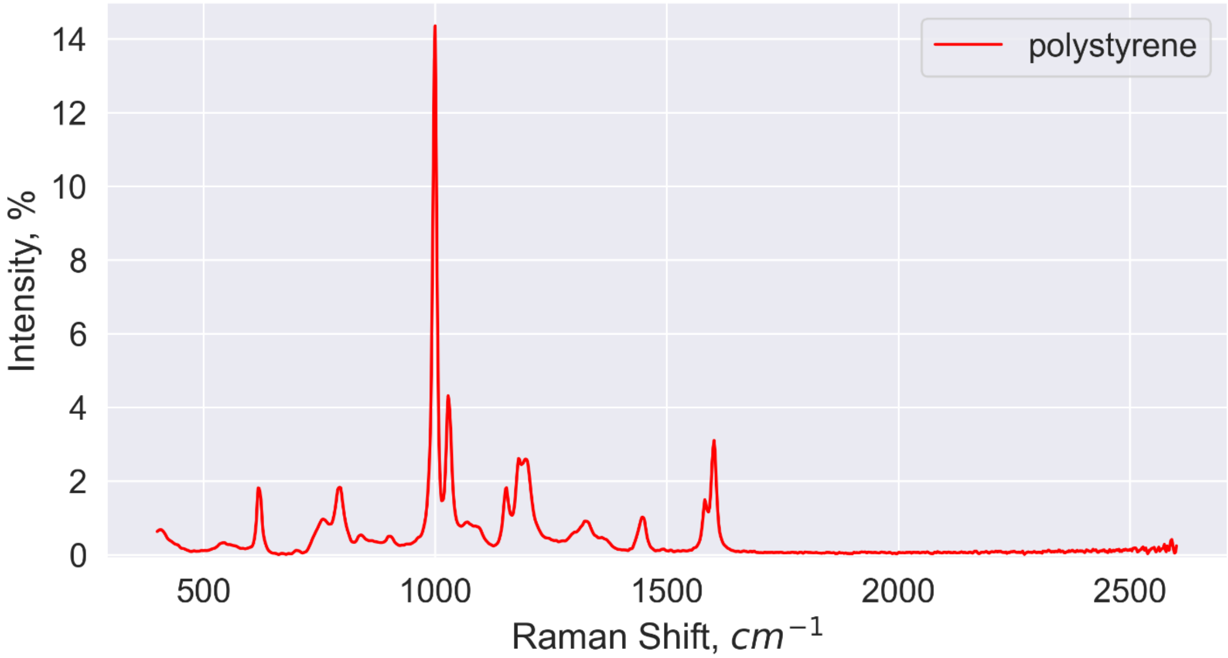

Fig. S15 Raman spectrum of polystyrene reference sample

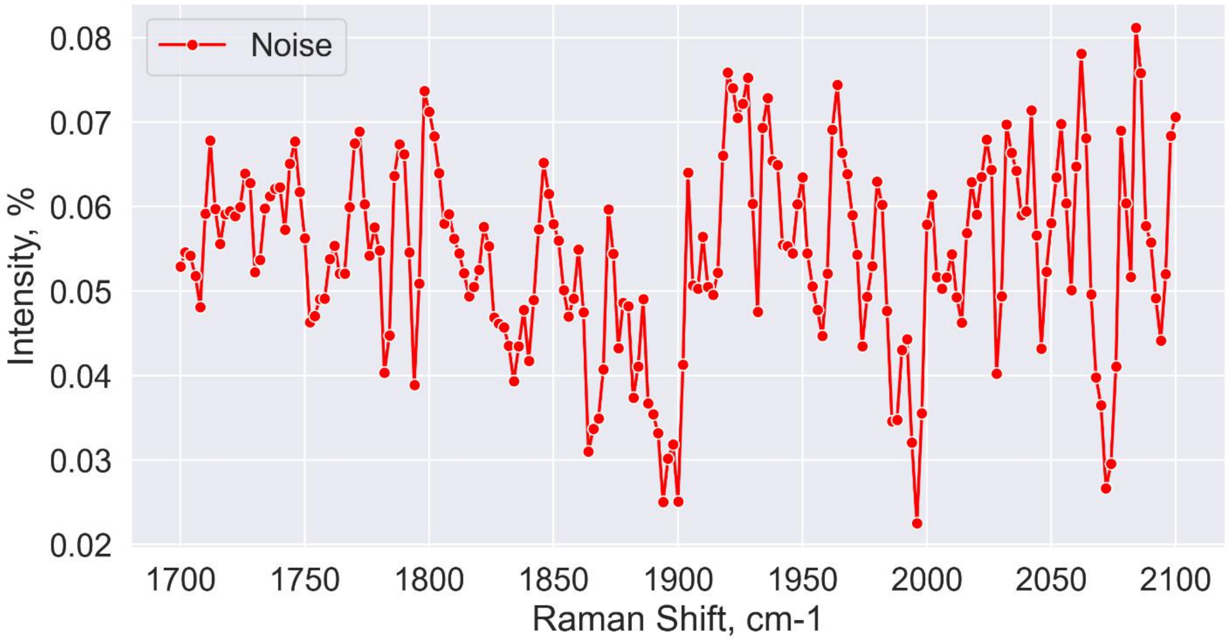

Fig. S16. Noise in the spectrum of polystyrene in the spectral range 1700..2100 cm-1

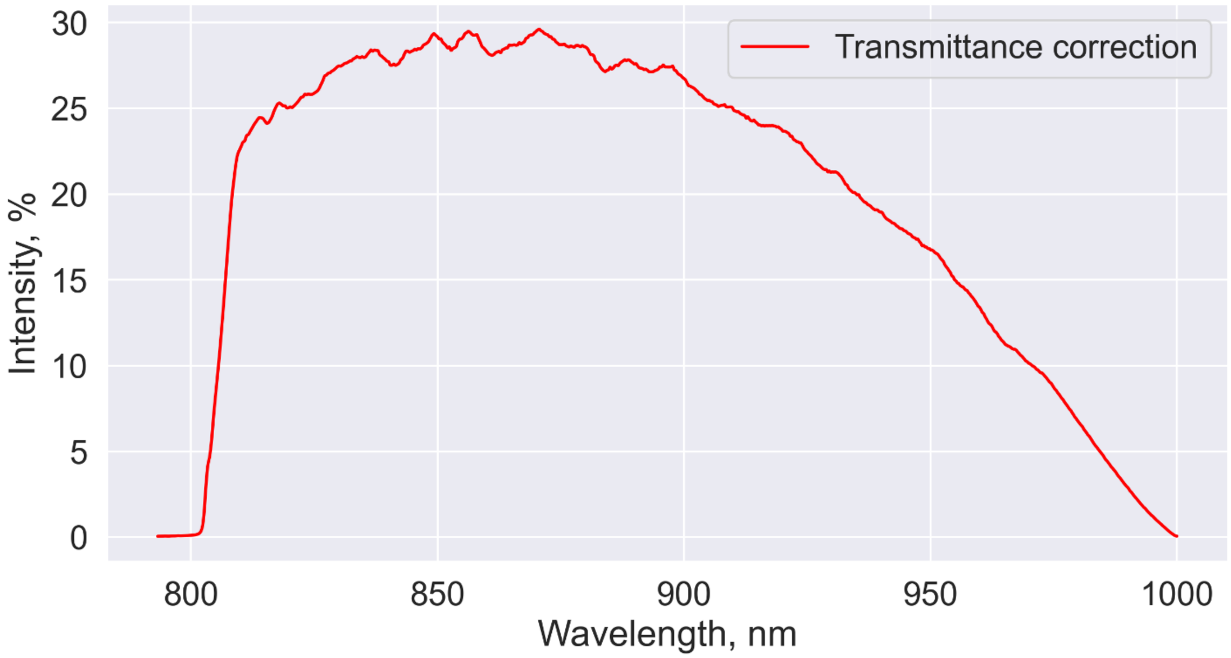

Fig. S17.Transmittance correction curve acquired with standard tungsten halogen source at 3000K

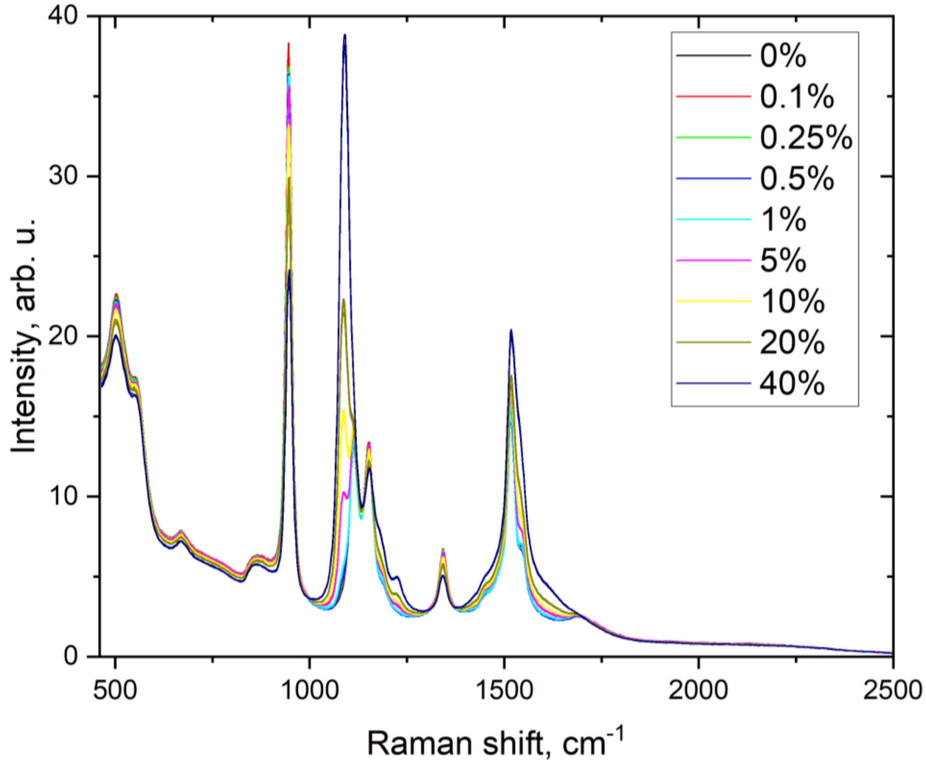

Fig. S18. Raman spectra of water ethanol solution with different concentration of methanol. Data obtained at laser excitation 785nm.

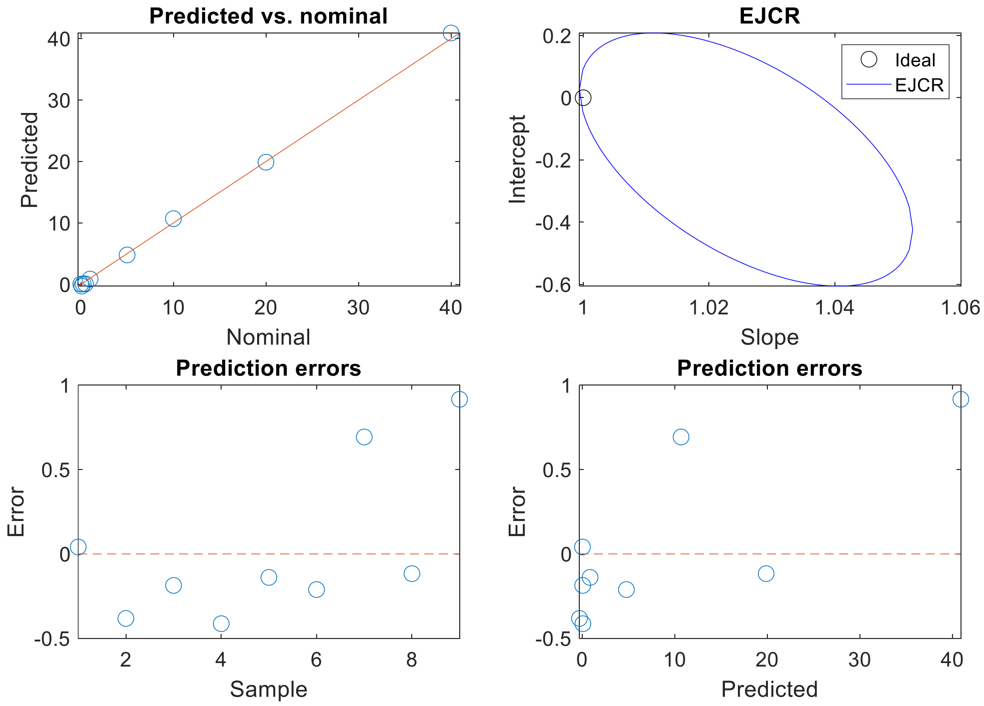

Fig. S19. Methanol quantification results obtained from laser excitation 785nm. Methanol detection was performed by PLS regression using MVC1 toolbox[4,5].

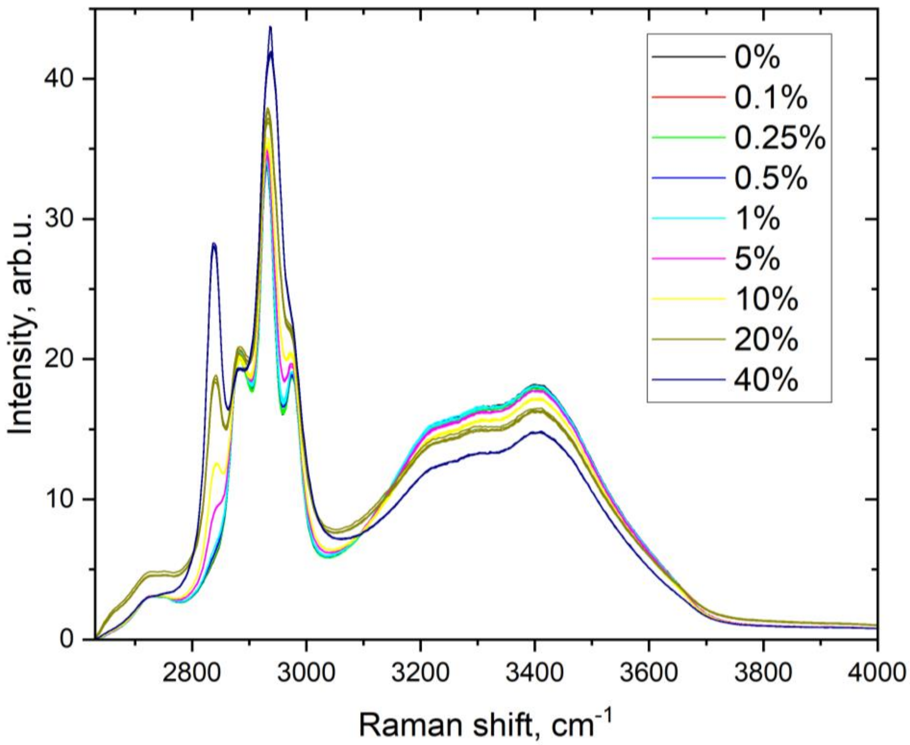

Fig. S209. Raman spectra of water ethanol solution with different concentration of methanol. Data obtained at laser excitation 675nm.

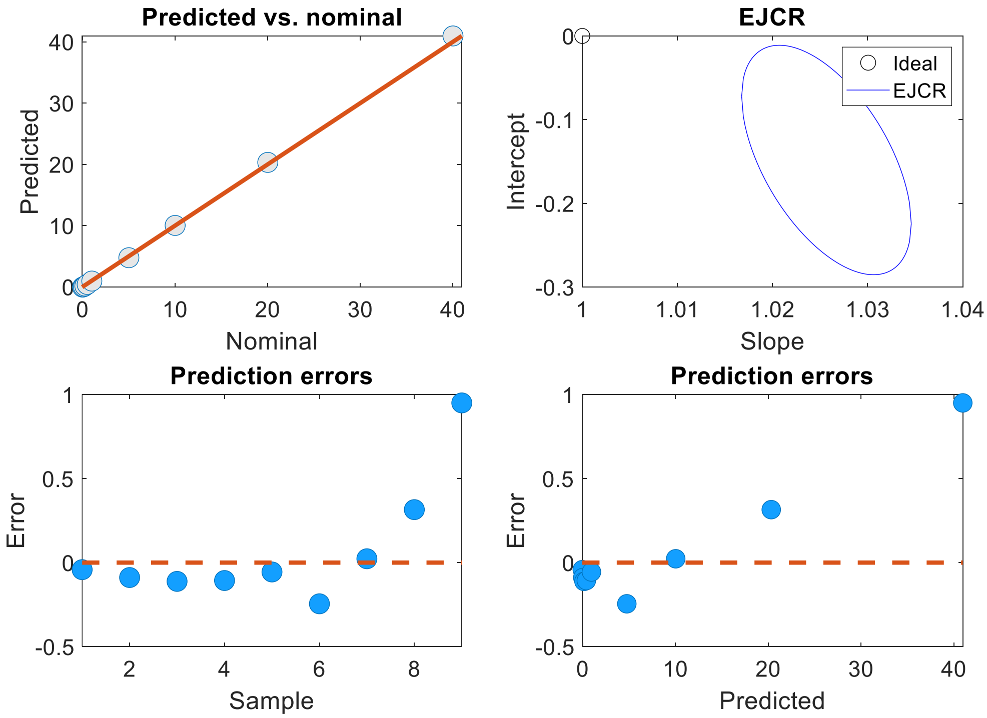

Fig. S21. Methanol quantification results obtained from laser excitation 675nm. Methanol detection was performed by PLS regression using MVC1 toolbox[4,5].

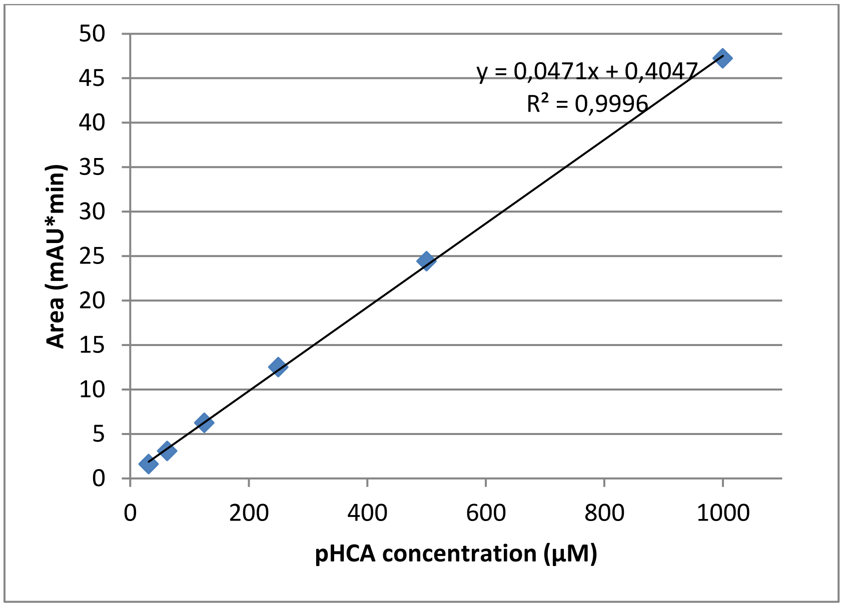

Fig. S22. PLS calibration based on Raman data for liquid samples of pHCA diluted in PBS

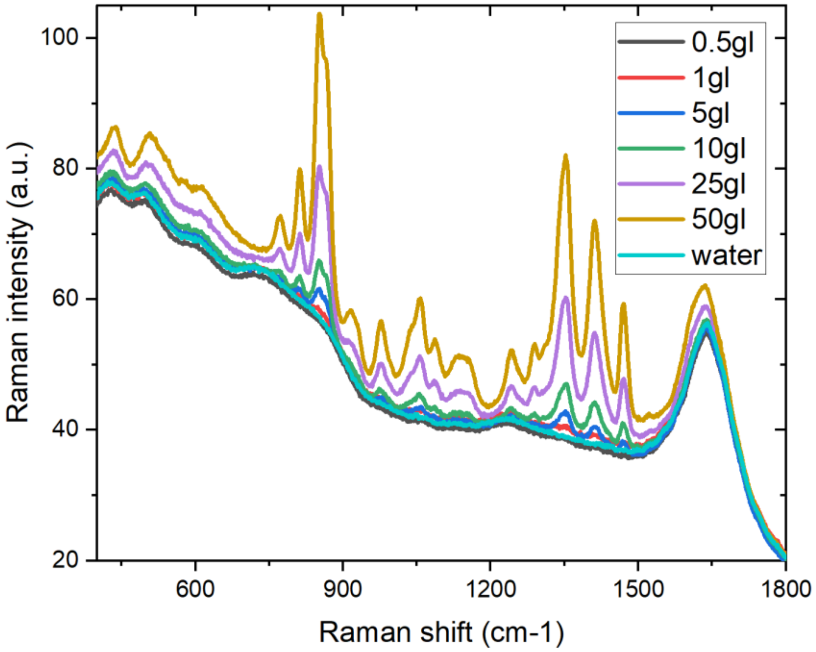

Fig. S23. Raman spectra of the different concentrations of Serine diluted in PBS

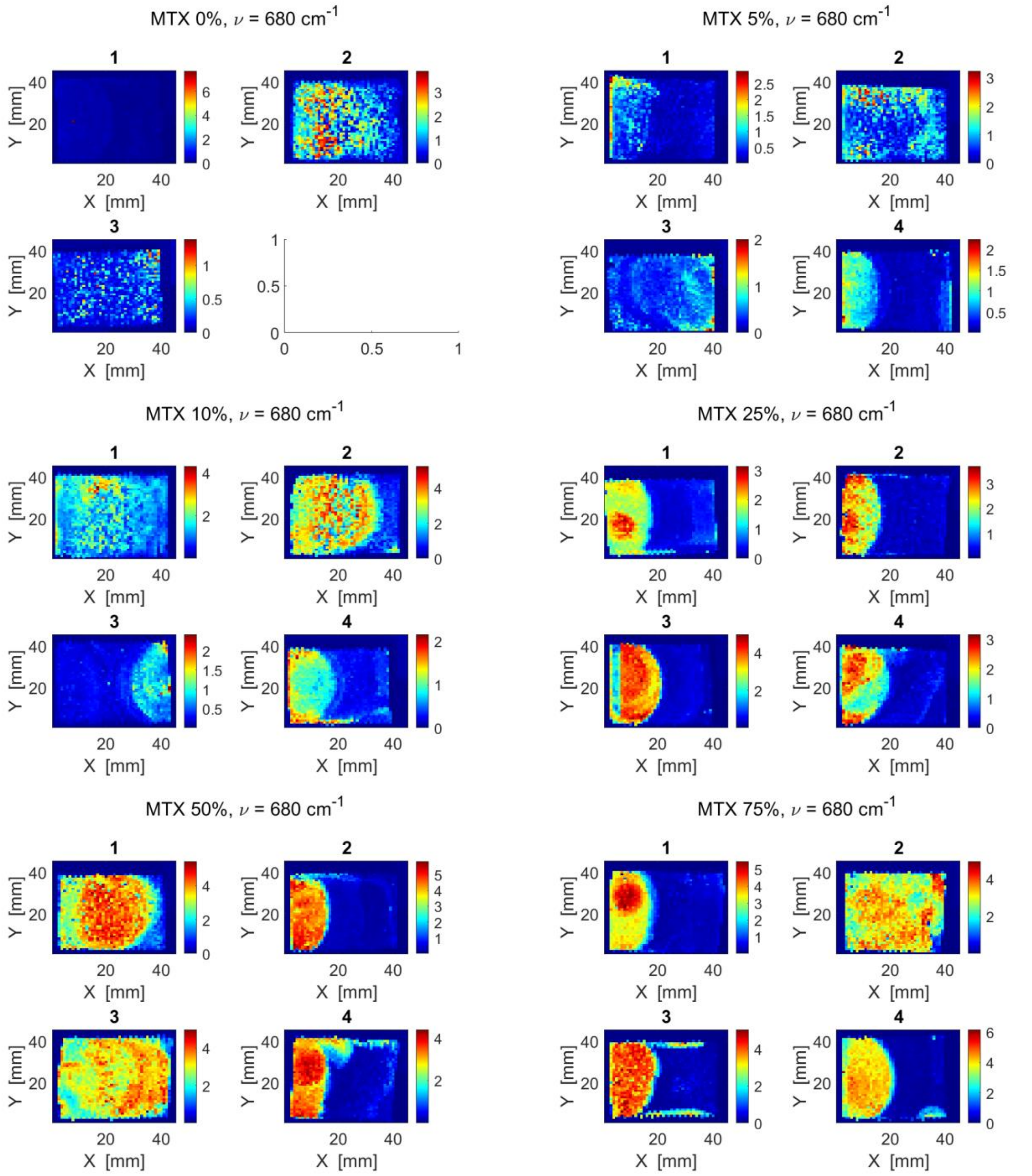

Fig. S24. Peak intensity spatial distribution of MTX

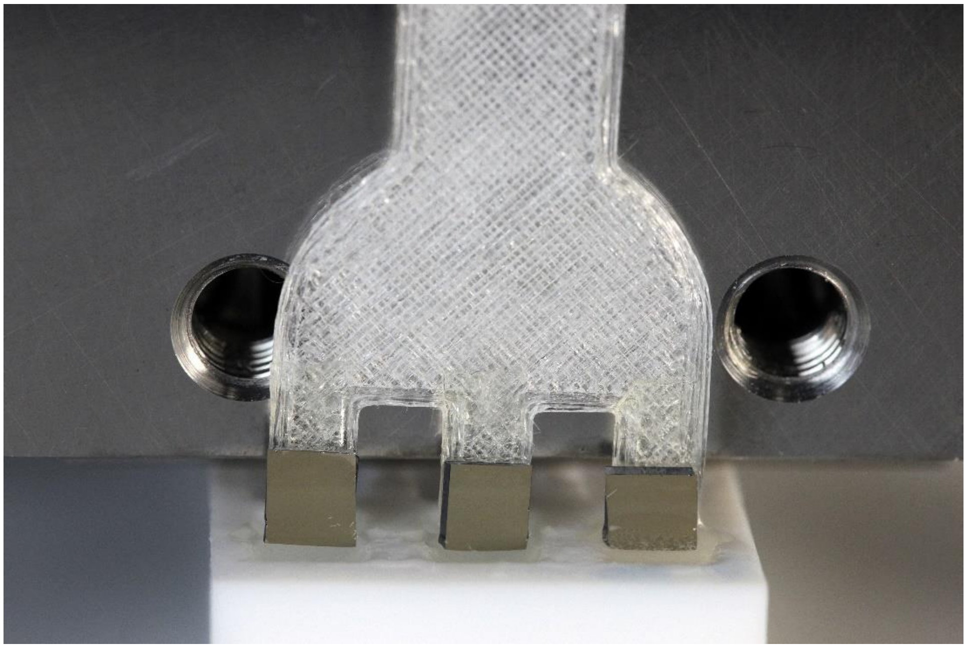

Fig. S25. Photograph of silver coated NP SERS chips inserted into solution with MTX.

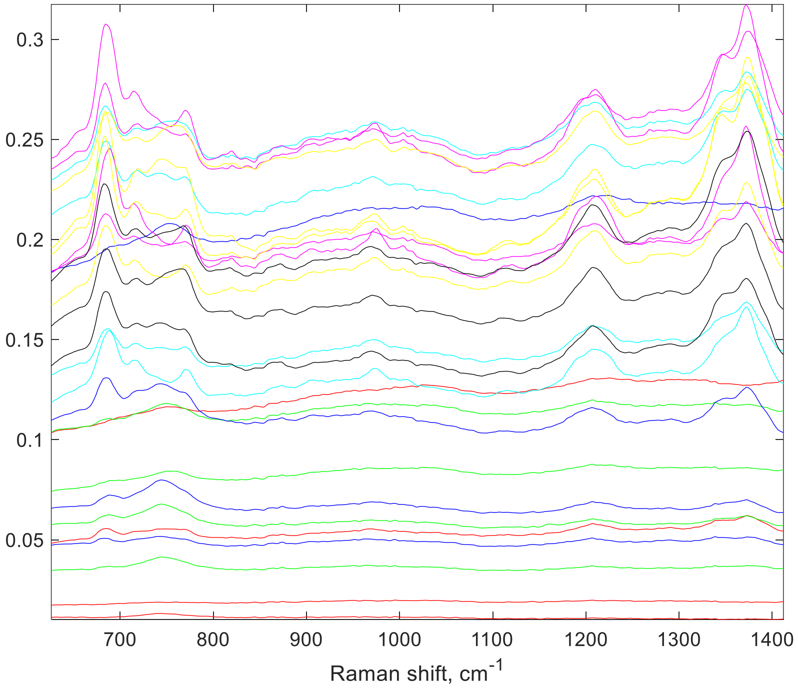

Fig. S26. Map averaged MTX spectra.

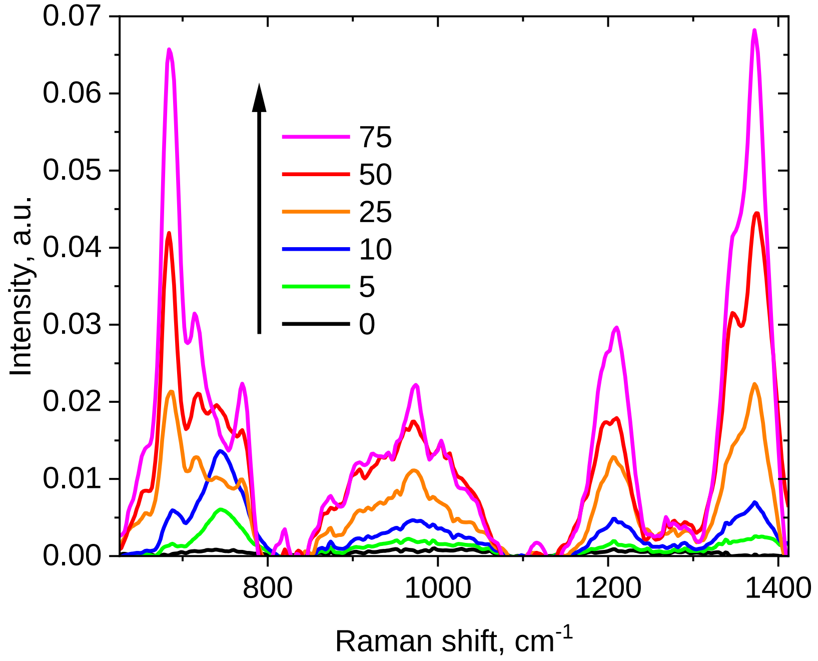

Fig. S27. Background corrected spectra. Background was eliminated using asymmetrical least squares with $\lambda = 10^5, p = 0.01$ with further mean-centering. Spectral range 626-1412 cm^{-1} was used for calibration.

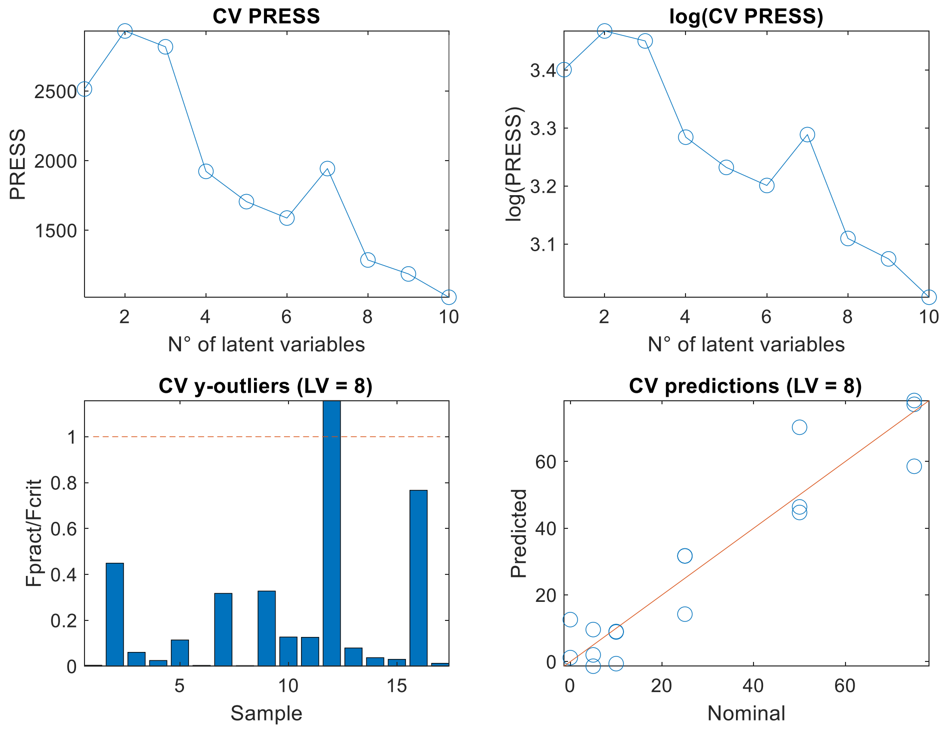

Fig. S28. MTX PLS calibration results. Analysis was performed by PLS regression using MVC1 toolbox[4,5].

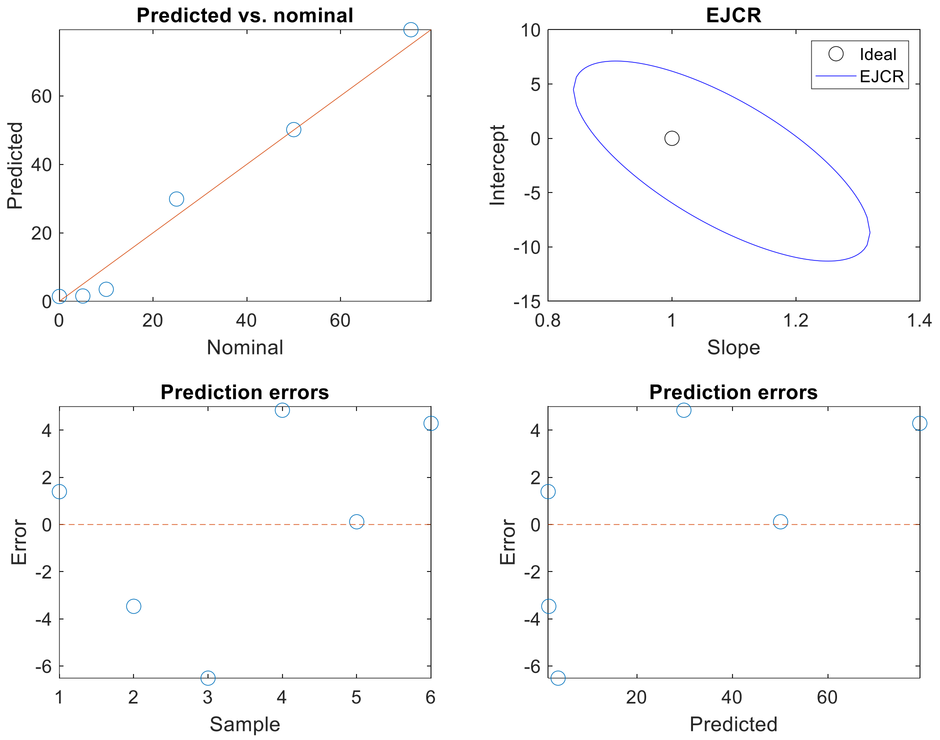

Fig. S29. MTX. PLS validaiton results. Analysis was performed by PLS regression using MVC1 toolbox[4,5].

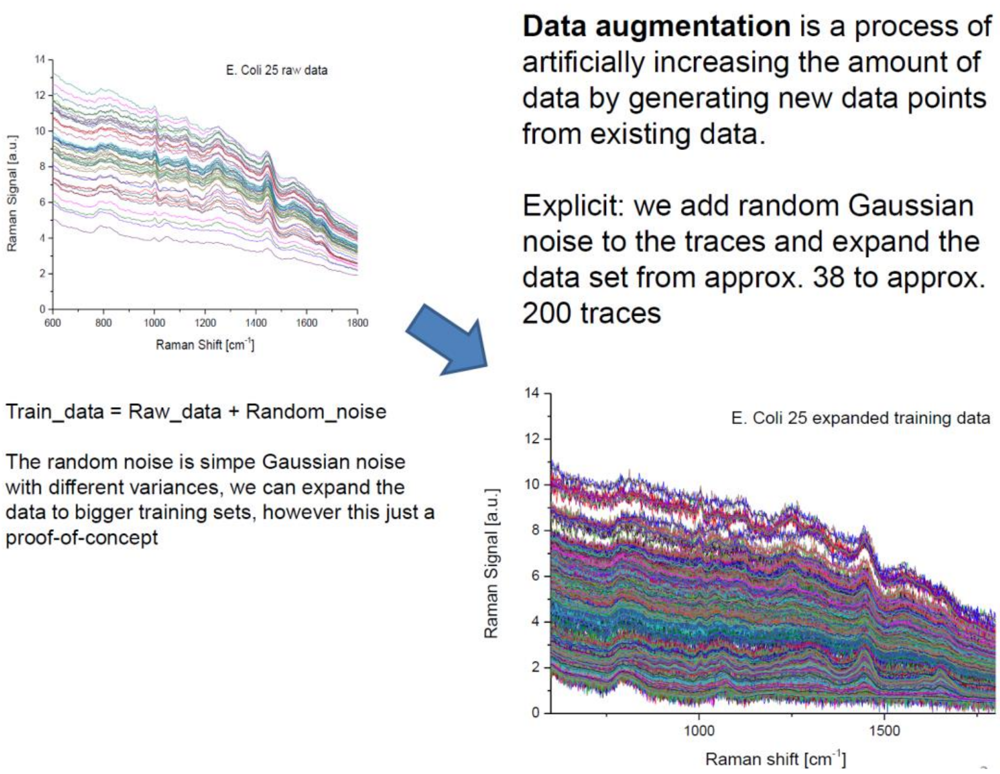

Fig. S30. ML of bacteria data

Spetrac Transformer							
	back CaF2	Ec 25	EC 35	MRSE	MSSE	Homo	Lug
back CaF2	40	0	0	0	0	0	0
Ec 25	0	37	0	2	0	1	0
EC 35	0	0	39	0	0	0	1
MRSE	7	0	0	40	0	0	1
MSSE	0	1	0	0	31	0	0
Homo	0	0	0	0	0	53	3
Lug	0	0	0	0	0	0	32
Accuracy = 100*Diagonal/total				94,44444			

CNN	ML						
	back CaF2	Ec 25	EC 35	MRSE	MSSE	Homo	Lug
back CaF2	40	0	0	0	0	0	0
Ec 25	0	38	0	1	1	0	0
EC 35	0	0	39	0	0	1	0
MRSE	0	0	0	47	0	1	0
MSSE	0	0	0	0	32	0	0
Homo	0	0	0	0	0	56	0
Lug	0	0	0	0	0	0	32
Accuracy = 100*Diagonal/total				98,61111			

Fig. S31. ML of bacteria data

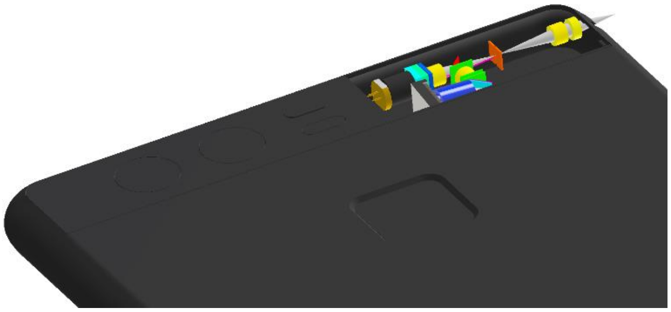

Fig. S32. The optomechanical design of miniaturized Raman spectrometer with spectral resolution 18cm-1 in the range 400-2000cm-1 operated from laser wavelength 785nm.

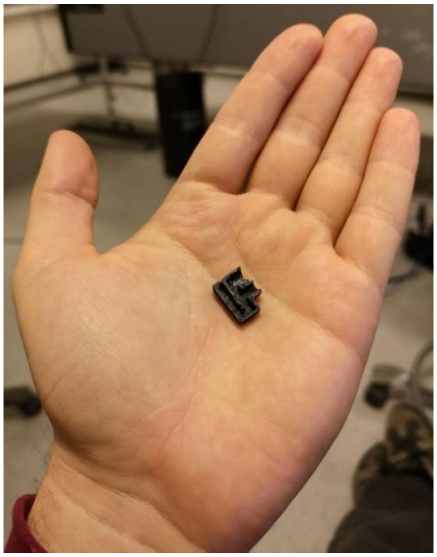

Fig. S33. Photograph of the prototype of miniaturized Raman spectrometer with spectral resolution 18cm-1 in the range 400-2000cm-1 operated from laser wavelength 785nm.

Tables

Table S1. **Methanol quantification from 785nm**

Parameter	Value
RMSECV	0.511
RMSEP	0.4411
R^2 prediction	0.998
LOD min	0.2746
LOD max	0.7739
LOQ min	0.8238
LOQ max	2.3217

Table S2. **Methanol quantification from 675nm**

Parameter	Value
RMSECV	0.1
RMSEP	0.4411
R^2 prediction	1.0
LOD min	0.0711
LOD max	0.1607
LOQ min	0.2134
LOQ max	0.4822

Table S3. pHCA calibration Raman

pHCA concentration (µM)	1	2	average	std dev
1000	47.2105	47.2451	47.2278	0.024466
500	24.4478	24.402	24.4249	0.032385
250	12.546	12.5231	12.53455	0.016193
125	6.278	6.2729	6.27545	0.003606
62.5	3.10259	3.102102	3.102346	0.000345
31.25	1.6234	1.6227	1.62305	0.000495

Table S4. Serine calibration Raman

g/L	Intensity	Intensity	Intensity
50	102	103	105
25	56	55.5	56
12.5	28	29	28.5
6.25	16	17	16.8
1	5	5.8	5.3

Table S5. **MTX quantification by SERS**

RMSECV	8.7
RMSEP	4.0475
R2 pred	0.9931
LOD min	2.9842
LOD max	6.875
LOQ min	8.9527
LOQ max	20.6251

Table S6. A comparison of the key Raman spectrometer and Raman microscope parameters for proposed miniaturized design concept, typical handheld and benchtop Raman spectrometer and typical high-end Raman microscope.

Device parameters	Proposed miniaturized design concept	Typical handheld Raman spectrometer	Typical benchtop Raman spectrometer	Typical high-end Raman microscope
**Raman spectrometer operation mode**				
Laser wavelength	785nm			
Laser power	~100mW			
Spectral resolution*	7-10cm^{-1}	7-15cm^{-1}*****	3-10cm^{-1}*****	1-7cm^{-1}*****
Spectral range	400-2600cm^{-1} (from 785nm) and 2600-4000cm^{-1} (from 675nm)	400-2500cm^{-1}*****	200-3200cm^{-1}*****	50-3200cm^{-1} (from 785nm)*****
SNR**	1256:1	~ 500:1	~ 1500:1	~ 1500:1
Laser temperature stabilization	Not required	Required	Required	Required
Operation readiness time***	5 sec	2-3 min	2-3 min	2-5 min
Wavenumber calibration periodicity	Every acquisition	Every month	Every month	Every year
Wavenumber calibration accuracy****	±1.5cm^{-1}	±2.5cm^{-1}	±1.5cm^{-1}	±1.5cm^{-1}
Laser power calibration	Every acquisition	Typically, no	Typically, no	Typically, no
SORS (Fig. 2j)	Yes	Typically, no	Typically, no	No
SERDS (Fig. 1n-1q)	Yes	Typically, no	Typically, no	No
Correction on pixel QE variation (Fig 2e-2g)	Yes	No	No	No
Reduction of dark noise via spectrum acquisition in a single row (Fig. 2a)	Yes	No	No	Typically, no
Operation time vs battery size*****	2 hours/1000mAh	2 hours/5000mAh	N/A	N/A
Optical part dimensions/ weight*****	(7x2x0.8cm)/(50g)	(20x15x3cm)/(500g)	(300x200x100cm)/(5kg)	(400x300x100cm)/(10k g)
Total dimensions/ weight*****	(11.2x3.9x3.4cm)/ (200g)	~(25x20x5cm)/(2kg)	~(400x300x200cm) /(10kg)	~(600x500x500cm)/(70 kg)

Materials cost****	~ €2.000	~ €7.000	~ €15.000	~ €30.000
**Raman microscope operation mode**				
Lateral resolution*****	Diffraction limited	N/A	N/A	Diffraction limited
Axial resolution*****	Diffraction limited	N/A	N/A	Diffraction limited
Dimensions, weight*****	(12x13x28cm)/(9kg)	N/A	N/A	~(600x500x500cm)/(70 kg)
Materials cost*****	~ €10.000	N/A	N/A	~ €70.000

*Spectral resolution of proposed miniaturized design concept of the Raman spectrometer was measured according to ASTM procedure (ASTM E2529 – 06(2014)) on Ca_2CO_3 sample, see details in Figure S14.
**SNR of proposed miniaturized design concept of the Raman spectrometer was determined as peak signal to noise ration of polystyrene spectrum at laser power 100mW, exposure time 0.3s, number of repetitions 10. SNRs for typical handheld, benchtop Raman spectrometers and high-end Raman microscopes were estimated based on Raman data of polystyrene spectra provided in several manuscripts[6–9]
***Operation readiness time determined as time between turning on the electronics and ability to collect accurate Raman spectrum (typically this time allocated for laser temperature stabilization)
****According to EUROPEAN PHARMACOPOEIA 10.7, 2.2.48. RAMAN SPECTROSCOPY, 04/2022:20248, p. 6360
*****Values for portable and benchtop Raman spectrometers estimated based on data provided by BCC Research "Global Markets for Raman Spectroscopy", Markets and Markets Research "Raman Spectrosocpy Market by Type, Instrument, Sampling Technique, Application and Region to 2028" and several review manuscripts[10–13], other values for Raman microscopes estimated based on review manuscript[14]

References

1. Eilers, P. H. C. Penalties in Chemometrics. in *Encyclopedia of Analytical Chemistry* 1–12 (Wiley, 2020). doi:10.1002/9780470027318.a9658.
2. Baek, S.-J., Park, A., Ahn, Y.-J. & Choo, J. Baseline correction using asymmetrically reweighted penalized least squares smoothing. *Analyst* **140**, 250–257 (2015).
3. Gazzola, S., Hansen, P. C. & Nagy, J. G. IR Tools: a MATLAB package of iterative regularization methods and large-scale test problems. *Numer. Algorithms* **81**, 773–811 (2019).
4. Olivieri, A. C., Goicoechea, H. C. & Iñón, F. A. MVC1: an integrated MatLab toolbox for first-order multivariate calibration. *Chemom. Intell. Lab. Syst.* **73**, 189–197 (2004).
5. Olivieri, A. C. Chemometrics and Multivariate Calibration. in *Introduction to Multivariate Calibration* 1–17 (Springer International Publishing, 2018). doi:10.1007/978-3-319-97097-4_1.
6. Ouillon, R. & Adam, S. Two accurate methods to obtain the spectral sensitivity of a Raman spectrometer device. *J. Raman Spectrosc.* **12**, 281–286 (1982).
7. Strola, S. A. *et al.* Single bacteria identification by Raman spectroscopy. *J. Biomed. Opt.* **19**, 111610 (2014).
8. Lu, J., Xue, Q., Bai, H. & Wang, N. Design of a confocal micro-Raman spectroscopy system and research on microplastics detection. *Appl. Opt.* **60**, 8375 (2021).
9. Fryling, M., Frank, C. J. & McCreery, R. L. Intensity Calibration and Sensitivity Comparisons for CCD/Raman Spectrometers. *Appl. Spectrosc.* **47**, 1965–1974 (1993).
10. Emmanuel, N., Nair, R. B., Abraham, B. & Yoosaf, K. Fabricating a Low-Cost Raman Spectrometer to Introduce Students to Spectroscopy Basics and Applied Instrument Design. *J. Chem. Educ.* **98**, 2109–2116 (2021).
11. Sing, D. *et al.* Design and demonstration of a portable and low-cost Raman spectrometer for rapid and low-cost estimation of marker molecules in plants. *Spectrosc. Lett.* **55**, 527–533 (2022).
12. DeGraff, B. A., Hennip, M., Jones, J. M., Salter, C. & Schaertel, S. A. An Inexpensive Laser Raman Spectrometer Based on CCD Detection. *Chem. Educ.* **7**, 15–18 (2002).
13. Bandyopadhyay, A. K., Dilawar, N., Vijayakumar, A., Varandani, D. & Singh, D. A low cost laser-raman spectrometer. *Bull. Mater. Sci.* **21**, 433–438 (1998).
14. Whitley, A. *et al.* Optimizing Depth Resolution in Confocal Raman Microscopy: A Comparison of Metallurgical, Dry Corrected, and Oil Immersion Objectives. *Appl. Spectrosc. Vol. 61, Issue 3, pp. 251-259* **61**, 251–259 (2007).
