# Supplementary material for: Optics miniaturization strategy for demanding Raman spectroscopy applications
Source: Nat Commun. 2024 Apr 8;15:3049. doi: 10.1038/s41467-024-47044-7 (PMC11001912; doi:10.1038/s41467-024-47044-7)
Supplement: Supplementary file 3 — Description of Additional Supplementary Files [file 41467_2024_47044_MOESM3_ESM.docx]

**Description of Additional Supplementary Files**

**Movie S1:** Demonstration of SORS performance in experiment of measuring whiskey content through the glass bottle
